# The Pay of the Nurse: III. The Value of Board and Lodging

**Published:** 1921-03-26

**Authors:** 


### THE MATRONS' AND SISTERS' DEPARTMENT.

### THE PAY OF THE NURSE.*

### III.—The Value of Board and Lodging.

Unquestionably one of the causes which is making nursing unpopular among the rising generation is that it so often entails living-in, or, in other words, receiving board and lodging as part payment for services. The advantages of being fed and housed free of cost are well known and often painted in glowing colours with all sincerity. It is claimed that nurses are far better housed, far better fed in institutions than in private life, that they are relieved from all domestic worries and provided with many interesting forms of recreation in community life.

These things are all perfectly true so far as nurses in training are concerned. But the estimate of their value to the nurse after her career has once fairly started is often misleading in the extreme. The value of food and lodging is no fixed sum. It is what it happens to be in the particular circumstances in which the nurse finds herself, and in the case of nurses doing private work these circumstances are continually varying. They may be landed in luxurious quarters where expensive delicacies are common adjuncts to the meal. Or they may have to make out their scanty and irregular rations with biscuits. They are not consulted and must take what is put before them without comment. It is much the same when they are housed in a nurses' institute. They cannot band together and run the commissariat on co-operative principles, paying their share of what it costs. They must accept what is given, and must bear to see a large proportion of their earnings spent for them by other people as though they were children, without having any voice in its disposition. The sum deducted from the salary for board and lodging is arbitrary. The thrifty woman who wishes to save is on the same level with the extravagant who has no sense of the value of money. Attempts to estimate it on the basis of what it would cost her to live outside are apt to mislead, for they can take no account of the immense difference in tastes, habits of living, and talent for economy.

What presses most hardly on the nurse compelled to live in (we are not here concerned with probationers) is the impediment the system places to the enjoyment of natural ties. The majority of women have relatives or friends with whom they can join up in any calling which does not involve living-in. By pooling their resources they can secure board and lodging at far lower terms than those estimated for the upkeep of an institute which requires the superintendence of a paid head. Were they paid a salary which did not include an arbitrary amount for board and lodging they might live at a rate far below that ordinarily computed, and in a manner more to their own taste. They cannot do this when board and lodging are supplied as part salary.

We find that from a pecuniary point of view non-residential posts are greatly to the advantage of the nurse. The College estimate of the minimum salary for non-residential posts is £250 per year, with allowance for uniform and an extra £10 for every required special qualification. In London or other large city the nurse would find that it cost her about £2 2s. a week for board and lodging (separate room), and about £10 for laundry. This works out at £120 a year, and leaves her with a clear salary of £130. In contrast with this we have the minimum for district nurses who live in estimated at £85, and it has been shown that private nurses seldom clear more than from £55 to £80. The non-residential nurse might, in some cases, be able to reduce her expenses by sharing a home with friends or by renting unfurnished rooms with attendance.

The tendency has been for the employer to over-estimate the value of board and lodging to the nurse, and hence to pare down the salaries of women who receive board as part payment for services. At one time it was even considered sufficient if it could be demonstrated that the nurse was able to live on her salary—that is, to keep herself in clothes and board herself during her fortnightly holiday. Always a movement has been perceptible in the direction of whittling down the earnings of those who live in, under the delusion that a woman can have no other expenses except those incurred in eating, sleeping, and clothing herself.

The drawbacks of the living-in system, which is compulsory on nurses in many branches of their profession, are that (1) it tends to lower salaries; (2) it impedes the nurse from the economy of joining forces with relatives or friends; (3) it eliminates just that spice of choice and variety in the daily fare which women specially appreciate; (4) it constantly forces upon them bad food, bad service, and galling restraint. A great part of the evils from which private and district nurses suffer come from an over-estimate of the value of the board and lodging they are compelled to accept.

### "The Coming of the Mob."

In our last issue appeared a vigorous and sincere protest from "Ierne" against the proposed admission to the Irish Register of existing nurses whose qualification rests on one year's hospital or Poor-law infirmary training. It is likely that it voices the feelings of many nurses, both in Ireland and in other parts of the United Kingdom, for although registration schemes have always involved the

* Previous articles appeared on March 5 and 12.

